# Misidentification of neural cell identity in liver-derived organoid systems

**DOI:** 10.1016/j.stemcr.2024.01.006

**Published:** 2024-02-08

**Authors:** Imre F. Schene, Arif I. Ardisasmita, Sabine A. Fuchs

**Affiliations:** 1 Department of Metabolic Diseases, Wilhelmina Children’s Hospital, University Medical Center Utrecht, Lundlaan 6, 3584 EA Utrecht, the Netherlands

Recently, an article titled “Platform-agnostic CellNet enables cross-study analysis of cell fate engineering protocols” was published in *Stem Cell Reports* (Lo et al., 2023). The authors developed an easy-to-use cell identity analysis using whole-transcriptome sequencing (RNA-seq) data called PACNet. We read the article with great interest and appreciate the development of PACNet, which allows for seamless cross-study analysis. However, we do not agree with one of the article’s main highlights, which states that “primary liver-derived organoids exhibit consistent off-target neural signatures.”

The authors observed the neural identity in the analysis of the RNA-seq datasets from Hu et al. (2018), Giobbe et al. (2019), Artegiani et al. (2019), and Akbari et al. (2019; Lo et al., 2023, Figure 6A), thereby including primary adult hepatocytes (PAHs), primary fetal hepatocytes (PFHs), fetal hepatocyte-derived organoids (fHOs), adult hepatocyte-derived organoids, liver-derived organoids (also known as intrahepatic cholangiocyte organoids [ICOs]), and pluripotent stem cell-derived liver organoids. While Lo et al. (2023) state that all these diverse samples exhibit a similar neural identity, we believe that the observed neural identity in these liver-related samples is an artifact caused by the processing of the data, specifically due to combining CEL-Seq2 library preparation with Salmon mapping.

First, we noticed that the presence of the neural identity did not correlate with sample type, but rather with the use of CEL-Seq2 for library preparation (Figure S1A). For example, PAHs and PFHs from Hu et al. (2018) have low liver and high neural classification scores as opposed to PAHs from studies that did not use CEL-Seq2, such as Xie et al. (2019; NEBNext) and Schneeberger et al. (2020; +NEXTflex). Lo et al. (2023) attribute this discrepancy to “inadvertent misannotation of some organoid samples as “primary”. However, when analyzing the samples from Hu et al. based on the count matrices available in the GEO repository (GSE111301), we found highly distinct expression profiles between sample types, showing correct sample annotation (Figures S1B and S1C).

Second, Lo et al. identify a neural identity in ICOs of Hu et al. (2018) and Artegiani et al. (2019; CEL-Seq2) but not in ICOs of Schneeberger et al. (2020; NEXTflex). Lo et al. suggest that the presence of neural identity in ICOs from specific studies is due to differences in culture protocols. However, the ICOs from Schneeberger et al., Hu et al., and Artegiani et al. were all established in Utrecht, using the same protocol developed by Huch et al. (2015).

To investigate the cause of the unexpected neural identity in the samples of Akbari et al., Artegiani et al., Giobbe et al., and Hu et al., we compiled the expression matrices provided by each author in the GEO repository (Akbari: GSE120145; Artegiani: GSE129457; Giobbe: GSE138611; Hu: GSE111301), and ran the PACNet analysis ourselves. Interestingly, our classification results (Figure S1D) differed from those reported in the original article (Lo et al., 2023). In our analysis, all samples from the studies that employed the CEL-Seq2 protocol did not exhibit a neural identity. Furthermore, the PAHs and PFHs of Hu et al. were correctly classified as liver, displaying liver classification scores similar to the PAHs and PFHs of Xie et al. Finally, all ICOs from the studies of Giobbe et al., Hu et al., and Artegiani et al. exhibited intestinal and liver identity, similar to the ICOs from Schneeberger et al.

We hypothesized that the discrepancies between Lo et al.’s analysis and our reanalysis was caused by different input of the gene expression matrices. Possibly, Lo et al. did not use the count matrices from the GEO repository, generated through a Burrows-Wheeler aligner, because they were not available as “preprocessed gene-by-sample expression matrices.” Instead, they may have applied Salmon mapping to CEL-Seq2-generated data. To test this, we generated count matrices for selected samples from the Hu et al., Artegiani et al., and Giobbe et al. studies through Salmon mapping on the Galaxy web platform, confirming that this altered classification scores, specifically decreasing liver identity and increasing neural identity (Figure S1E).

Having shown that the neural identity in liver-derived organoid systems is overestimated in the original article, we then focused on neuronal markers (NCAM1, SOX1, and NEUROD1) that were putatively expressed in ICOs (Lo et al., 2023, Figures 6B–6E). To investigate the expression of these markers, we referred to transcriptional data from our cross-study comparison of various hepatocyte *in vitro* models (Ardisasmita et al., 2022). In this work, we encountered problems integrating data from studies that employed the CEL-Seq2 protocol. For instance, the sample types from Hu et al. (e.g., PAHs, PFHs, and ICOs) did not cluster with the same sample types from other studies that did not employ the CEL-Seq2 protocol. This led us to recommend to always run internal primary tissue controls to validate compatibility for cross-study RNA-seq analysis. Besides, it led us to collaborate with the groups of Artegiani and Hu to generate new and compatible RNA-seq data of ICOs, fHOs, and primary tissue controls. Using these datasets, we found that SOX1 and NEUROD1 were not expressed in any liver-derived organoid system. In fact, expression of these genes was higher in PSC-derived liver models (Figure S1F). In addition, analysis of GEO-deposited count matrices of ICOs from the groups of Artegiani, Giobbe, Hu, and Schneeberger (GSE123498) confirmed the lack of expression of NCAM1, SOX1, or NEUROD1 (Figure S1G).

The lack of expression of SOX1 and NEUROD1 in ICOs seems to conflict with the immunostaining of these proteins in the original article (Lo et al., 2023). We suspect that the cytoplasmic SOX1 and NEUROD1 stainings represent background stainings, since typical stainings are nuclear (Figure S1H), and no negative control samples or western blot validations were provided.

In conclusion, we do not believe that liver-derived organoid systems exhibit neural identity. Our reevaluation indicates that the neural identity may rather relate to sample processing of CEL-Seq2 data combined with Salmon mapping. Moreover, to ensure valid classification of query samples and comparisons across studies using PACNet, we recommend consistent use of internal primary tissue controls.

## Supplemental information

Document S1. Figure S1

Document S2. Article plus supplemental information
