# Supplementary material for: Misidentification of neural cell identity in liver-derived organoid systems
Source: Stem Cell Reports. 2024 Feb 8;19(3):315–6. doi: 10.1016/j.stemcr.2024.01.006 (PMC10937148; doi:10.1016/j.stemcr.2024.01.006)
Supplement: Document S1. Figure S1 [file mmc1.pdf]

Stem Cell Reports, Volume *19*

Supplemental Information

Misidentification of neural cell identity in liver-derived organoid systems

Imre F. Schene, Arif I. Ardisasmita, and Sabine A. Fuchs

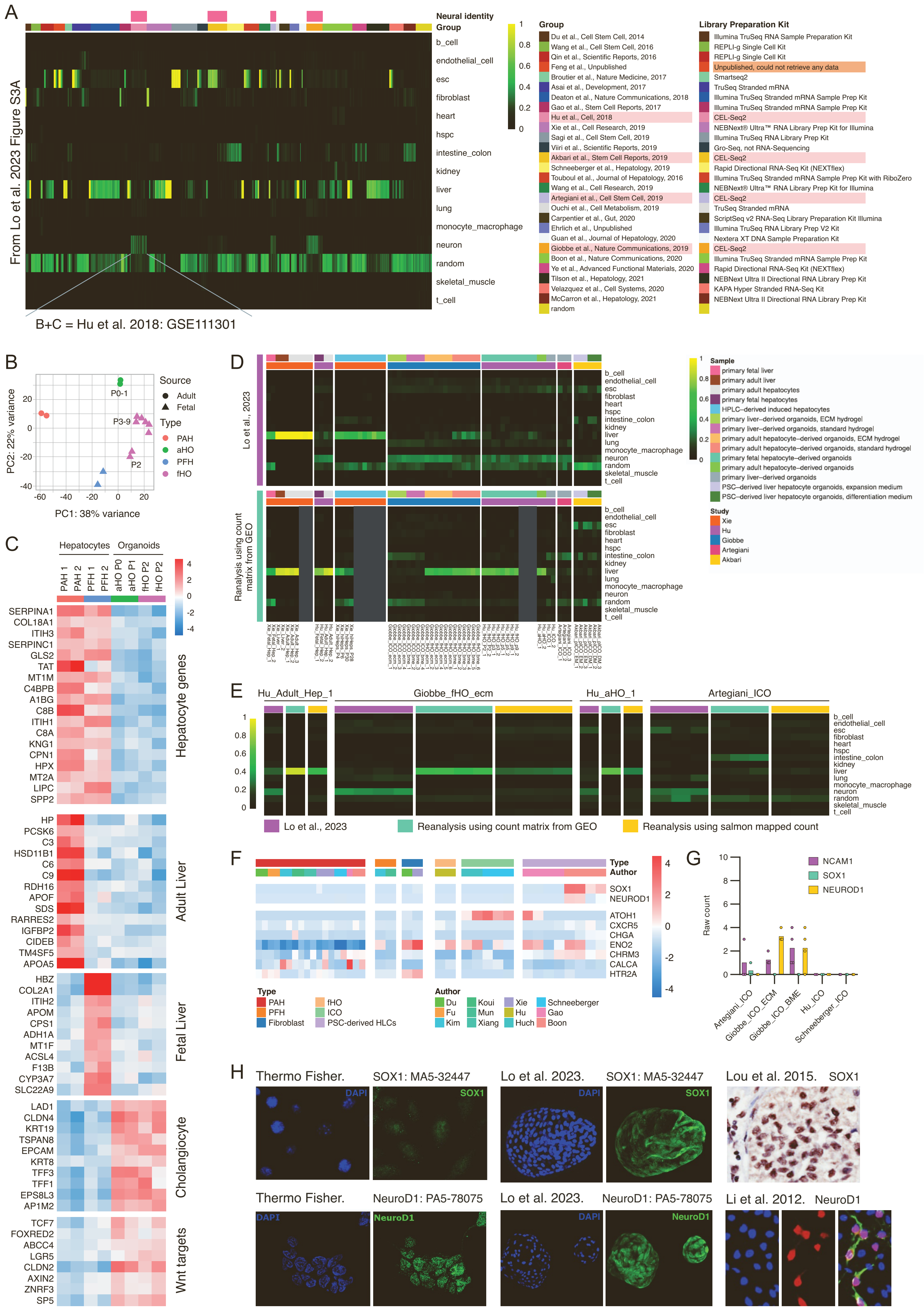
A
From Lo et al. 2023 Figure S3A
Neural identity
Group
b_cell
endothelial_cell
esc
fibroblast
heart
hspc
intestine_colon
kidney
liver
lung
monocyte_macrophage
neuron
random
skeletal_muscle
t_cell
B+C = Hu et al. 2018: GSE111301
Group
Du et al., Cell Stem Cell, 2014
Wang et al., Cell Stem Cell, 2016
Qin et al., Scientific Reports, 2016
Feng et al., Unpublished
Broutier et al., Nature Medicine, 2017
Asai et al., Development, 2017
Deaton et al., Nature Communications, 2018
Gao et al., Stem Cell Reports, 2017
Hu et al., Cell, 2018
Xie et al., Cell Research, 2019
Sagi et al., Cell Stem Cell, 2019
Viiri et al., Scientific Reports, 2019
Akbari et al., Stem Cell Reports, 2019
Schneeberger et al., Hepatology, 2019
Touboul et al., Journal of Hepatology, 2016
Wang et al., Cell Research, 2019
Artegiani et al., Cell Stem Cell, 2019
Ouchi et al., Cell Metabolism, 2019
Carpentier et al., Gut, 2020
Ehrlich et al., Unpublished
Guan et al., Journal of Hepatology, 2020
Giobbe et al., Nature Communications, 2019
Boon et al., Nature Communications, 2020
Ye et al., Advanced Functional Materials, 2020
Tilson et al., Hepatology, 2021
Velazquez et al., Cell Systems, 2020
McCarron et al., Hepatology, 2021
random
Library Preparation Kit
Illumina TruSeq RNA Sample Preparation Kit
REPLI-g Single Cell Kit
REPLI-g Single Cell Kit
Unpublished, could not retrieve any data
Smartseq2
TruSeq Stranded mRNA
Illumina TruSeq Stranded mRNA Sample Prep Kit
Illumina TruSeq Stranded mRNA Sample Prep Kit
CEL-Seq2
NEBNext® Ultra™ RNA Library Prep Kit for Illumina
Illumina TruSeq RNA Library Prep Kit
Gro-Seq, not RNA-Sequencing
CEL-Seq2
Rapid Directional RNA-Seq Kit (NEXTflex)
Illumina TruSeq Stranded mRNA Sample Prep Kit with RiboZero
NEBNext® Ultra™ RNA Library Prep Kit for Illumina
CEL-Seq2
TruSeq Stranded mRNA
ScriptSeq v2 RNA-Seq Library Preparation Kit Illumina
Illumina TruSeq RNA Library Prep V2 Kit
Nextera XT DNA Sample Preparation Kit
CEL-Seq2
Illumina TruSeq Stranded mRNA Sample Prep Kit
Rapid Directional RNA-Seq Kit (NEXTflex)
NEBNext Ultra II Directional RNA Library Prep Kit
KAPA Hyper Stranded RNA-Seq Kit
NEBNext Ultra II Directional RNA Library Prep Kit
B
PC2: 22% variance
PC1: 38% variance
P0-1
P3-9
P2
Source
Adult
Fetal
Type
PAH
aHO
PFH
fHO
C
Hepatocytes
Organoids
PAH 1
PAH 2
PFH 1
PFH 2
aHO P0
aHO P1
fHO P2
fHO P2
Hepatocyte genes
SERPINA1
COL18A1
ITIH3
SERPINC1
GLS2
TAT
MT1M
C4BPB
A1BG
C8B
ITIH1
C8A
KNG1
CPN1
HPX
MT2A
LIPC
SPP2
Adult Liver
HP
PCSK6
C3
HSD11B1
C6
C9
RDH16
APOF
SDS
RARRES2
IGFBP2
CIDEB
TM4SF5
APOA5
Fetal Liver
HBZ
COL2A1
ITIH2
APOM
CPS1
ADH1A
MT1F
ACSL4
F13B
CYP3A7
SLC22A9
Cholangiocyte
LAD1
CLDN4
KRT19
TSPAN8
EPCAM
KRT8
TFF3
TFF1
EPS8L3
AP1M2
Wnt targets
TCF7
FOXRED2
ABCC4
LGR5
CLDN2
AXIN2
ZNRF3
SP5
D
Lo et al., 2023
Ranalysis using count matrix from GEO
Sample
primary fetal liver
primary adult liver
primary adult hepatocytes
primary fetal hepatocytes
HPLC–derived induced hepatocytes
primary liver–derived organoids, ECM hydrogel
primary liver–derived organoids, standard hydrogel
primary adult hepatocyte–derived organoids, ECM hydrogel
primary adult hepatocyte–derived organoids, standard hydrogel
primary fetal hepatocyte–derived organoids
primary adult hepatocyte–derived organoids
primary liver–derived organoids
PSC–derived liver hepatocyte organoids, expansion medium
PSC–derived liver hepatocyte organoids, differentiation medium
Study
Xie
Hu
Giobbe
Artegiani
Akbari
E
Hu_Adult_Hep_1
Giobbe_fHO_ecm
Hu_aHO_1
Artegiani_ICO
Lo et al., 2023
Reanalysis using count matrix from GEO
Reanalysis using salmon mapped count
F
Type
Author
SOX1
NEUROD1
ATOH1
CXCR5
CHGA
ENO2
CHRM3
CALCA
HTR2A
Type
PAH
PFH
Fibroblast
fHO
ICO
PSC-derived HLCs
Author
Du
Fu
Kim
Koui
Mun
Xiang
Xie
Hu
Huch
Schneeberger
Gao
Boon
G
Raw count
NCAM1
SOX1
NEUROD1
Artegiani_ICO
Giobbe_ICO_ECM
Giobbe_ICO_BME
Hu_ICO
Schneeberger_ICO
H
Thermo Fisher.
SOX1: MA5-32447
Lo et al. 2023.
SOX1: MA5-32447
Lou et al. 2015.
SOX1
DAPI
SOX1
Thermo Fisher.
NeuroD1: PA5-78075
Lo et al. 2023.
NeuroD1: PA5-78075
Li et al. 2012.
NeuroD1
NeuroD1

Figure S1. Liver-derived organoid systems do not exhibit neural identity.

(A) Classification heatmap from the original Lo et al. (2023, Figure S3A) study showing that only samples from the studies of Hu, Akbari, Artegiani, and Giobbe have neural identity. The right column represents the library preparation protocols for all RNA-Seq data and indicates that only these four studies employed CEL-Seq2. ESC = embryonic stem cells; HSPC = hematopoietic stem and progenitor cells.

(B) Principal component plot of selected samples from the Hu et al. (2018) study, showing that primary adult and fetal hepatocytes (PAH, PFH) do not cluster with hepatic organoids.

(C) Heatmaps of selected genes from the samples in (B). High expression of adult and fetal liver markers in PAHs and PFHs, respectively, combined with low expression of typical liver organoid markers (cholangiocyte and Wnt-target genes) refutes incorrect sample labeling as a possible cause for neural identity in PAHs and PFHs from the study of Hu et al. (2018).

(D) Classification heatmaps from the Lo et al. (2023) study (top) and the same samples, reanalyzed by applying the PACNet web-based analysis on the count matrices as deposited in the GEO repository by the original studies (bottom). Note strikingly lower neural classification scores in the original, GEO-deposited data.

(E) Similar to (D), but only for selected samples and also including PACNet web-based analysis on the counts as generated by re-mapping of the CEL-Seq2-generated fastq-files using Salmon mapping on the Galaxy web platform (salmon_quant, usegalaxy.eu)

(F) Gene expression heatmaps of selected neural markers, generated by the HLCompR web interfaced and based on our cross-study comparison of liver models (Ardisasmita et al., 2022).

(G) Total counts of neural marker genes in all included ICO studies. Note that the figure represents counts as reported in the GEO repository by the original studies, indicating virtual absence of expression of NCAM1, SOX1, and NEUROD1.

(H) Comparison of immunostaining of SOX1 (top) and NeuroD1 (bottom) as reported by the manufacturer of the antibodies (left panels; nuclear staining) and the Lo et al. (2023) study (middle panels, cytoplasmic staining). Note that other studies into SOX1 and NeuroD1 also report nuclear staining (right panels).
