# Supplementary material for: Misidentification of neural cell identity in liver-derived organoid systems
Source: Stem Cell Reports. 2024 Feb 8;19(3):315–6. doi: 10.1016/j.stemcr.2024.01.006 (PMC10937148; doi:10.1016/j.stemcr.2024.01.006)
Supplement: Document S2. Article plus supplemental information [file mmc2.pdf]

Stem Cell Reports
Letter
OPEN ACCESS

Misidentification of neural cell identity in liver-derived organoid systems

Imre F. Schene,[1,2] Arif I. Ardisasmita,[1,2] and Sabine A. Fuchs[1,*]
[1]Department of Metabolic Diseases, Wilhelmina Children's Hospital, University Medical Center Utrecht, Lundlaan 6, 3584 EA Utrecht, the Netherlands
[2]These authors contributed equally
*Correspondence: s.fuchs@umcutrecht.nl
https://doi.org/10.1016/j.stemcr.2024.01.006

Recently, an article titled "Platform-agnostic CellNet enables cross-study analysis of cell fate engineering protocols" was published in *Stem Cell Reports* (Lo et al., 2023). The authors developed an easy-to-use cell identity analysis using whole-transcriptome sequencing (RNA-seq) data called PACNet. We read the article with great interest and appreciate the development of PACNet, which allows for seamless cross-study analysis. However, we do not agree with one of the article's main highlights, which states that "primary liver-derived organoids exhibit consistent off-target neural signatures."

The authors observed the neural identity in the analysis of the RNA-seq datasets from Hu et al. (2018), Giobbe et al. (2019), Artegiani et al. (2019), and Akbari et al. (2019; Lo et al., 2023, Figure 6A), thereby including primary adult hepatocytes (PAHs), primary fetal hepatocytes (PFHs), fetal hepatocyte-derived organoids (fHOs), adult hepatocyte-derived organoids, liver-derived organoids (also known as intrahepatic cholangiocyte organoids [ICOs]), and pluripotent stem cell-derived liver organoids. While Lo et al. (2023) state that all these diverse samples exhibit a similar neural identity, we believe that the observed neural identity in these liver-related samples is an artifact caused by the processing of the data, specifically due to combining CEL-Seq2 library preparation with Salmon mapping.

First, we noticed that the presence of the neural identity did not correlate with sample type, but rather with the use of CEL-Seq2 for library preparation (Figure S1A). For example, PAHs and PFHs from Hu et al. (2018) have low liver and high neural classification scores as opposed to PAHs from studies that did not use CEL-Seq2, such as Xie et al. (2019; NEBNext) and Schneeberger et al. (2020; +NEXTflex). Lo et al. (2023) attribute this discrepancy to "inadvertent misannotation of some organoid samples as "primary". However, when analyzing the samples from Hu et al. based on the count matrices available in the GEO repository (GSE111301), we found highly distinct expression profiles between sample types, showing correct sample annotation (Figures S1B and S1C).

Second, Lo et al. identify a neural identity in ICOs of Hu et al. (2018) and Artegiani et al. (2019; CEL-Seq2) but not in ICOs of Schneeberger et al. (2020; NEXTflex). Lo et al. suggest that the presence of neural identity in ICOs from specific studies is due to differences in culture protocols. However, the ICOs from Schneeberger et al., Hu et al., and Artegiani et al. were all established in Utrecht, using the same protocol developed by Huch et al. (2015).

To investigate the cause of the unexpected neural identity in the samples of Akbari et al., Artegiani et al., Giobbe et al., and Hu et al., we compiled the expression matrices provided by each author in the GEO repository (Akbari: GSE120145; Artegiani: GSE129457; Giobbe: GSE138611; Hu: GSE111301), and ran the PACNet analysis ourselves. Interestingly, our classification results (Figure S1D) differed from those reported in the original article (Lo et al., 2023). In our analysis, all samples from the studies that employed the CEL-Seq2 protocol did not exhibit a neural identity. Furthermore, the PAHs and PFHs of Hu et al. were correctly classified as liver, displaying liver classification scores similar to the PAHs and PFHs of Xie et al. Finally, all ICOs from the studies of Giobbe et al., Hu et al., and Artegiani et al. exhibited intestinal and liver identity, similar to the ICOs from Schneeberger et al.

We hypothesized that the discrepancies between Lo et al.'s analysis and our reanalysis was caused by different input of the gene expression matrices. Possibly, Lo et al. did not use the count matrices from the GEO repository, generated through a Burrows-Wheeler aligner, because they were not available as "preprocessed gene-by-sample expression matrices." Instead, they may have applied Salmon mapping to CEL-Seq2-generated data. To test this, we generated count matrices for selected samples from the Hu et al., Artegiani et al., and Giobbe et al. studies through Salmon mapping on the Galaxy web platform, confirming that this altered classification scores, specifically decreasing liver identity and increasing neural identity (Figure S1E).

Having shown that the neural identity in liver-derived organoid systems is overestimated in the original article, we then focused on neuronal markers (NCAM1, SOX1, and NEUROD1) that were putatively expressed in ICOs (Lo et al., 2023, Figures 6B–6E). To investigate the expression of these markers, we referred to transcriptional data from our cross-study comparison of various hepatocyte *in vitro* models (Ardisasmita et al., 2022). In this work, we encountered problems integrating data from studies that employed the CEL-Seq2 protocol. For instance, the sample

This is an open access article under the CC BY license (http://creativecommons.org/licenses/by/4.0/).

types from Hu et al. (e.g., PAHs, PFHs, and ICOs) did not cluster with the same sample types from other studies that did not employ the CEL-Seq2 protocol. This led us to recommend to always run internal primary tissue controls to validate compatibility for cross-study RNA-seq analysis. Besides, it led us to collaborate with the groups of Artegiani and Hu to generate new and compatible RNA-seq data of ICOs, fHOs, and primary tissue controls. Using these datasets, we found that SOX1 and NEUROD1 were not expressed in any liver-derived organoid system. In fact, expression of these genes was higher in PSC-derived liver models (Figure S1F). In addition, analysis of GEO-deposited count matrices of ICOs from the groups of Artegiani, Giobbe, Hu, and Schneeberger (GSE123498) confirmed the lack of expression of NCAM1, SOX1, or NEUROD1 (Figure S1G).

The lack of expression of SOX1 and NEUROD1 in ICOs seems to conflict with the immunostaining of these proteins in the original article (Lo et al., 2023). We suspect that the cytoplasmic SOX1 and NEUROD1 stainings represent background stainings, since typical stainings are nuclear (Figure S1H), and no negative control samples or western blot validations were provided.

In conclusion, we do not believe that liver-derived organoid systems exhibit neural identity. Our reevaluation indicates that the neural identity may rather relate to sample processing of CEL-Seq2 data combined with Salmon mapping. Moreover, to ensure valid classification of query samples and comparisons across studies using PACNet, we recommend consistent use of internal primary tissue controls.

SUPPLEMENTAL INFORMATION

Supplemental information can be found online at https://doi.org/10.1016/j.stemcr.2024.01.006.

ACKNOWLEDGMENTS

This study was supported by ZonMW TAS (“Regenerating Intestinal Tissue with Stem cells” project) and Stichting WKZ Fonds.

DECLARATION OF INTERESTS

The authors declare no competing interests.

REFERENCES

Akbari, S., Sevinç, G.G., Ersoy, N., Basak, O., Kaplan, K., Sevinç, K., Ozel, E., Sengun, B., Enustun, E., Ozcimen, B., et al. (2019). Robust, Long-Term Culture of Endoderm-Derived Hepatic Organoids for Disease Modeling. Stem Cell Rep. *13*, 627–641.

Ardisasmita, A.I., Schene, I.F., Joore, I.P., Kok, G., Hendriks, D., Artegiani, B., Mokry, M., Nieuwenhuis, E.E.S., and Fuchs, S.A. (2022). A comprehensive transcriptomic comparison of hepatocyte model systems improves selection of models for experimental use. Commun. Biol. *5*.

Artegiani, B., van Voorthuijsen, L., Lindeboom, R.G.H., Seinstra, D., Heo, I., Tapia, P., López-Iglesias, C., Postrach, D., Dayton, T., Oka, R., et al. (2019). Probing the Tumor Suppressor Function of BAP1 in CRISPR-Engineered Human Liver Organoids. Cell Stem Cell *24*, 927–943.e6.

Giobbe, G.G., Crowley, C., Luni, C., Campinoti, S., Khedr, M., Kretzschmar, K., De Santis, M.M., Zambaiti, E., Michielin, F., Meran, L., et al. (2019). Extracellular matrix hydrogel derived from decellularized tissues enables endodermal organoid culture. Nat. Commun. *10*, 5658.

Hu, H., Gehart, H., Artegiani, B., LÖpez-Iglesias, C., Dekkers, F., Basak, O., van Es, J., Chuva de Sousa Lopes, S.M., Begthel, H., Korving, J., et al. (2018). Long-Term Expansion of Functional Mouse and Human Hepatocytes as 3D Organoids. Cell *175*, 1591–1606.e19.

Huch, M., Gehart, H., Van Boxtel, R., Hamer, K., Blokzijl, F., Verstegen, M.M.A., Ellis, E., Van Wenum, M., Fuchs, S.A., De Ligt, J., et al. (2015). Long-term culture of genome-stable bipotent stem cells from adult human liver. Cell *160*, 299–312.

Lo, E.K.W., Velazquez, J.J., Peng, D., Kwon, C., Ebrahimkhani, M.R., and Cahan, P. (2023). Platform-agnostic CellNet enables cross-study analysis of cell fate engineering protocols. Stem Cell Rep. *18*, 1721–1742.

Schneeberger, K., Sánchez-Romero, N., Ye, S., van Steenbeek, F.G., Oosterhoff, L.A., Pla Palacin, I., Chen, C., van Wolferen, M.E., van Tienderen, G., Lieshout, R., et al. (2020). Large-Scale Production of LGR5-Positive Bipotential Human Liver Stem Cells. Hepatology *72*, 257–270.

Xie, B., Sun, D., Du, Y., Jia, J., Sun, S., Xu, J., Liu, Y., Xiang, C., Chen, S., Xie, H., et al. (2019). A two-step lineage reprogramming strategy to generate functionally competent human hepatocytes from fibroblasts. Cell Res. *29*, 696–710.

Stem Cell Reports, Volume *19*

Supplemental Information

Misidentification of neural cell identity in liver-derived organoid systems

Imre F. Schene, Arif I. Ardisasmita, and Sabine A. Fuchs

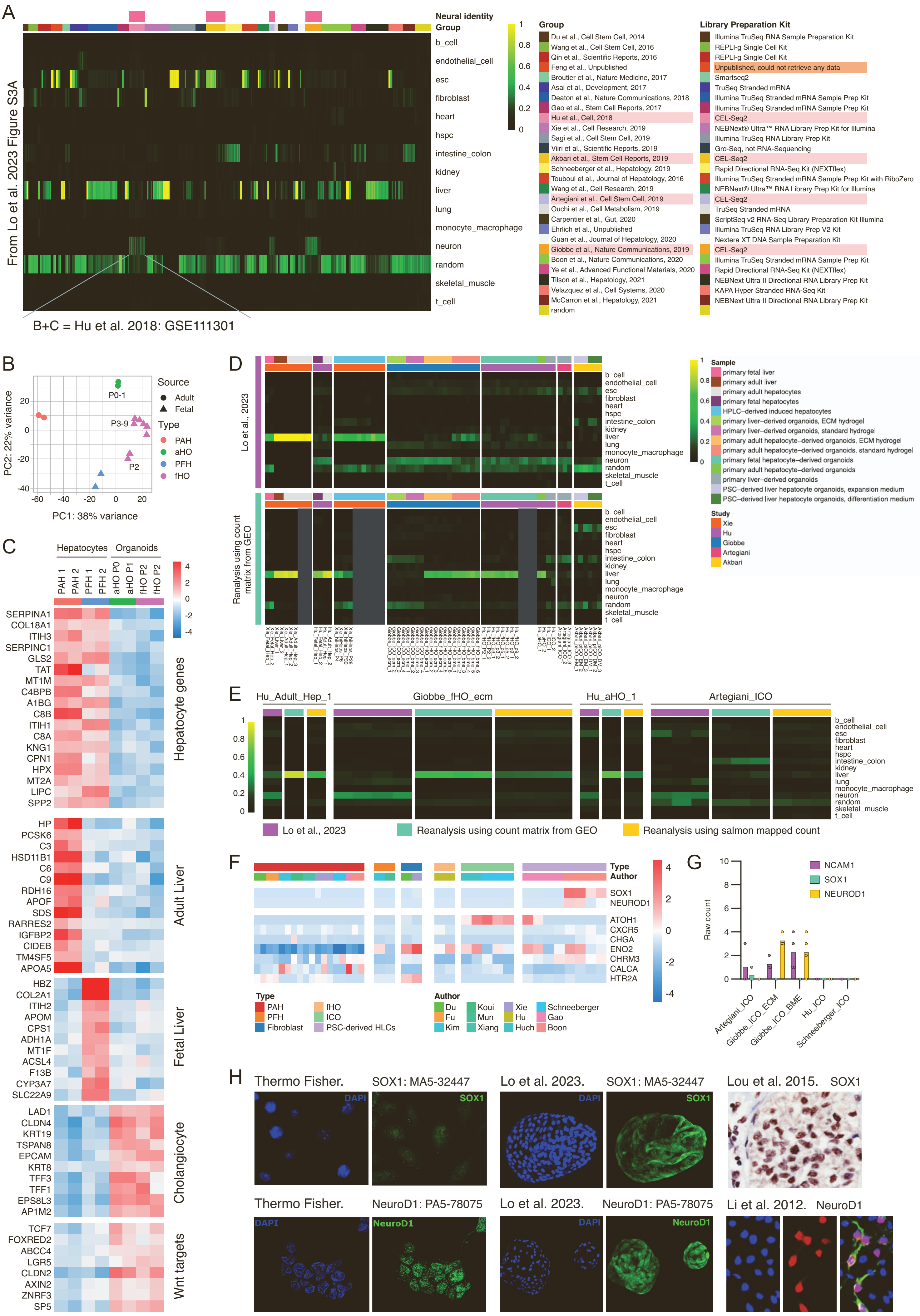
A
From Lo et al. 2023 Figure S3A
Neural identity
Group
b_cell
endothelial_cell
esc
fibroblast
heart
hspc
intestine_colon
kidney
liver
lung
monocyte_macrophage
neuron
random
skeletal_muscle
t_cell
1
0.8
0.6
0.4
0.2
0
B+C = Hu et al. 2018: GSE111301
Group
Du et al., Cell Stem Cell, 2014
Wang et al., Cell Stem Cell, 2016
Qin et al., Scientific Reports, 2016
Feng et al., Unpublished
Broutier et al., Nature Medicine, 2017
Asai et al., Development, 2017
Deaton et al., Nature Communications, 2018
Gao et al., Stem Cell Reports, 2017
Hu et al., Cell, 2018
Xie et al., Cell Research, 2019
Sagi et al., Cell Stem Cell, 2019
Viiri et al., Scientific Reports, 2019
Akbari et al., Stem Cell Reports, 2019
Schneeberger et al., Hepatology, 2019
Touboul et al., Journal of Hepatology, 2016
Wang et al., Cell Research, 2019
Artegiani et al., Cell Stem Cell, 2019
Ouchi et al., Cell Metabolism, 2019
Carpentier et al., Gut, 2020
Ehrlich et al., Unpublished
Guan et al., Journal of Hepatology, 2020
Giobbe et al., Nature Communications, 2019
Boon et al., Nature Communications, 2020
Ye et al., Advanced Functional Materials, 2020
Tilson et al., Hepatology, 2021
Velazquez et al., Cell Systems, 2020
McCarron et al., Hepatology, 2021
random
Library Preparation Kit
Illumina TruSeq RNA Sample Preparation Kit
REPLI-g Single Cell Kit
REPLI-g Single Cell Kit
Unpublished, could not retrieve any data
Smartseq2
TruSeq Stranded mRNA
Illumina TruSeq Stranded mRNA Sample Prep Kit
Illumina TruSeq Stranded mRNA Sample Prep Kit
CEL-Seq2
NEBNext® Ultra™ RNA Library Prep Kit for Illumina
Illumina TruSeq RNA Library Prep Kit
Gro-Seq, not RNA-Sequencing
CEL-Seq2
Rapid Directional RNA-Seq Kit (NEXTflex)
Illumina TruSeq Stranded mRNA Sample Prep Kit with RiboZero
NEBNext® Ultra™ RNA Library Prep Kit for Illumina
CEL-Seq2
TruSeq Stranded mRNA
ScriptSeq v2 RNA-Seq Library Preparation Kit Illumina
Illumina TruSeq RNA Library Prep V2 Kit
Nextera XT DNA Sample Preparation Kit
CEL-Seq2
Illumina TruSeq Stranded mRNA Sample Prep Kit
Rapid Directional RNA-Seq Kit (NEXTflex)
NEBNext Ultra II Directional RNA Library Prep Kit
KAPA Hyper Stranded RNA-Seq Kit
NEBNext Ultra II Directional RNA Library Prep Kit
B
PC2: 22% variance
PC1: 38% variance
20
0
-20
-40
-60
-40
-20
0
20
P0-1
P3-9
P2
Source
Adult
Fetal
Type
PAH
aHO
PFH
fHO
C
Hepatocytes
Organoids
PAH 1
PAH 2
PFH 1
PFH 2
aHO P0
aHO P1
fHO P2
fHO P2
4
2
0
-2
-4
SERPINA1
COL18A1
ITIH3
SERPINC1
GLS2
TAT
MT1M
C4BPB
A1BG
C8B
ITIH1
C8A
KNG1
CPN1
HPX
MT2A
LIPC
SPP2
Hepatocyte genes
HP
PCSK6
C3
HSD11B1
C6
C9
RDH16
APOF
SDS
RARRES2
IGFBP2
CIDEB
TM4SF5
APOA5
Adult Liver
HBZ
COL2A1
ITIH2
APOM
CPS1
ADH1A
MT1F
ACSL4
F13B
CYP3A7
SLC22A9
Fetal Liver
LAD1
CLDN4
KRT19
TSPAN8
EPCAM
KRT8
TFF3
TFF1
EPS8L3
AP1M2
Cholangiocyte
TCF7
FOXRED2
ABCC4
LGR5
CLDN2
AXIN2
ZNRF3
SP5
Wnt targets
D
Lo et al., 2023
Ranalysis using count matrix from GEO
b_cell
endothelial_cell
esc
fibroblast
heart
hspc
intestine_colon
kidney
liver
lung
monocyte_macrophage
neuron
random
skeletal_muscle
t_cell
Sample
primary fetal liver
primary adult liver
primary adult hepatocytes
primary fetal hepatocytes
HPLC–derived induced hepatocytes
primary liver–derived organoids, ECM hydrogel
primary liver–derived organoids, standard hydrogel
primary adult hepatocyte–derived organoids, ECM hydrogel
primary adult hepatocyte–derived organoids, standard hydrogel
primary fetal hepatocyte–derived organoids
primary adult hepatocyte–derived organoids
primary liver–derived organoids
PSC–derived liver hepatocyte organoids, expansion medium
PSC–derived liver hepatocyte organoids, differentiation medium
Study
Xie
Hu
Giobbe
Artegiani
Akbari
E
Hu_Adult_Hep_1
Giobbe_fHO_ecm
Hu_aHO_1
Artegiani_ICO
1
0.8
0.6
0.4
0.2
0
Lo et al., 2023
Reanalysis using count matrix from GEO
Reanalysis using salmon mapped count
F
Type
Author
SOX1
NEUROD1
ATOH1
CXCR5
CHGA
ENO2
CHRM3
CALCA
HTR2A
4
2
0
-2
-4
Type
PAH
PFH
Fibroblast
fHO
ICO
PSC-derived HLCs
Author
Du
Fu
Kim
Koui
Mun
Xiang
Xie
Hu
Huch
Schneeberger
Gao
Boon
G
Raw count
10
8
6
4
2
0
NCAM1
SOX1
NEUROD1
Artegiani_ICO
Giobbe_ICO_ECM
Giobbe_ICO_BME
Hu_ICO
Schneeberger_ICO
H
Thermo Fisher.
SOX1: MA5-32447
DAPI
SOX1
Lo et al. 2023.
SOX1: MA5-32447
DAPI
SOX1
Lou et al. 2015.
SOX1
Thermo Fisher.
NeuroD1: PA5-78075
DAPI
NeuroD1
Lo et al. 2023.
NeuroD1: PA5-78075
DAPI
NeuroD1
Li et al. 2012.
NeuroD1

Figure S1. Liver-derived organoid systems do not exhibit neural identity.

(A) Classification heatmap from the original Lo et al. (2023, Figure S3A) study showing that only samples from the studies of Hu, Akbari, Artegiani, and Giobbe have neural identity. The right column represents the library preparation protocols for all RNA-Seq data and indicates that only these four studies employed CEL-Seq2. ESC = embryonic stem cells; HSPC = hematopoietic stem and progenitor cells.

(B) Principal component plot of selected samples from the Hu et al. (2018) study, showing that primary adult and fetal hepatocytes (PAH, PFH) do not cluster with hepatic organoids.

(C) Heatmaps of selected genes from the samples in (B). High expression of adult and fetal liver markers in PAHs and PFHs, respectively, combined with low expression of typical liver organoid markers (cholangiocyte and Wnt-target genes) refutes incorrect sample labeling as a possible cause for neural identity in PAHs and PFHs from the study of Hu et al. (2018).

(D) Classification heatmaps from the Lo et al. (2023) study (top) and the same samples, reanalyzed by applying the PACNet web-based analysis on the count matrices as deposited in the GEO repository by the original studies (bottom). Note strikingly lower neural classification scores in the original, GEO-deposited data.

(E) Similar to (D), but only for selected samples and also including PACNet web-based analysis on the counts as generated by re-mapping of the CEL-Seq2-generated fastq-files using Salmon mapping on the Galaxy web platform (salmon_quant, usegalaxy.eu)

(F) Gene expression heatmaps of selected neural markers, generated by the HLCompR web interfaced and based on our cross-study comparison of liver models (Ardisasmita et al., 2022).

(G) Total counts of neural marker genes in all included ICO studies. Note that the figure represents counts as reported in the GEO repository by the original studies, indicating virtual absence of expression of NCAM1, SOX1, and NEUROD1.

(H) Comparison of immunostaining of SOX1 (top) and NeuroD1 (bottom) as reported by the manufacturer of the antibodies (left panels; nuclear staining) and the Lo et al. (2023) study (middle panels, cytoplasmic staining). Note that other studies into SOX1 and NeuroD1 also report nuclear staining (right panels).
